# Clinical effectiveness and safety of baricitinib for the treatment of juvenile idiopathic arthritis-associated uveitis or chronic anterior antinuclear antibody-positive uveitis: study protocol for an open-label, adalimumab active-controlled phase 3 clinical trial (JUVE-BRIGHT)

**DOI:** 10.1186/s13063-021-05651-5

**Published:** 2021-10-09

**Authors:** Athimalaipet V. Ramanan, Catherine M. Guly, Stuart Y. Keller, Douglas E. Schlichting, Stephanie de Bono, Ran Liao, Pierre Quartier

**Affiliations:** 1 Translational Health Sciences, University of Bristol, Bristol, UK; 2 Department of Paediatric Rheumatology, University Hospitals Bristol NHS Foundation Trust, Bristol Royal Hospital for Children, Upper Maudlin Street, Bristol, BS2 8BJ UK; 3 Bristol Eye Hospital, University Hospitals Bristol NHS Foundation Trust, Bristol, UK; 4 Eli Lilly and Company, Indianapolis, IN USA; 5 Pediatric Immunology-Hematology and Rheumatology Unit, RAISE reference centre for rare diseases, Necker-Enfants Malades University Hospital, Assistance Publique-Hopitaux de Paris, IMAGINE Institute, Université de Paris, Paris, France

**Keywords:** Antinuclear antibody-positive, Baricitinib, Bayesian analysis, Juvenile idiopathic arthritis, Open-label Bayesian design, Ophthalmology, Pediatric, Randomized controlled trials, Rheumatology, Uveitis

## Abstract

**Background:**

Juvenile idiopathic arthritis (JIA) is the most common pediatric rheumatic disease and the most common systemic disorder associated with uveitis in childhood. Uveitis is more common in JIA patients who are antinuclear antibody (ANA)-positive, have an early-onset disease, and have oligoarticular arthritis. JIA-associated uveitis (JIA-uveitis) is typically anterior, chronic, bilateral, nongranulomatous, and asymptomatic. Visual outcomes in JIA-uveitis have improved with current screening and treatment options; however, many patients fail to respond or do not achieve long-lasting remission. Baricitinib, an oral selective Janus kinase (JAK)1 and 2 inhibitor, may impact key cytokines implicated in the pathogenesis of JIA-uveitis or ANA-positive uveitis, representing a potential novel treatment option for disease management.

**Methods:**

The multicenter, phase 3 trial will be conducted using an open-label Bayesian design. The study will enroll at least 20 and up to 40 patients aged 2 to <18 years with active JIA-uveitis or chronic ANA-positive uveitis without systemic features. At least 20 patients who have had an inadequate response or intolerance to methotrexate (MTX-IR), but not biologic disease-modifying antirheumatic drugs (bDMARDs), will be randomized (1:1) to open-label baricitinib or adalimumab. Approximately 20 additional patients who are MTX-IR or bDMARD inadequate responders will receive baricitinib treatment. Patients will be treated with once daily oral baricitinib at a fixed dose by age group (4 mg for patients aged ≥6 to <18 years and 2 mg for patients <6 years) or adalimumab (20 mg for patients weighing <30 kg and 40 mg for patients ≥30 kg) as a subcutaneous injection every 2 weeks. Treatment with stable background conventional synthetic DMARDs, low-dose corticosteroids, and/or nonsteroidal anti-inflammatory drugs is allowed. The primary endpoint is the proportion of patients with response at week 24. Patients may continue treatment for up to 5 years.

**Discussion:**

This is the first pediatric clinical trial to assess the clinical effectiveness and safety of a JAK inhibitor in JIA-uveitis or chronic ANA-positive uveitis. A novel Bayesian design is used to assess the efficacy of baricitinib, including an adalimumab reference arm, in this small patient population with unmet medical need.

**Trial registration:**

EudraCT 2019-000119-10. Registered on January 4, 2019; NCT04088409. Registered on September 12, 2019

## Administrative information

Note: the numbers in curly brackets in this protocol refer to SPIRIT checklist item numbers. The order of the items has been modified to group similar items (see http://www.equator-network.org/reporting-guidelines/spirit-2013-statement-defining-standard-protocol-items-for-clinical-trials/).

**Table:** 

Title {1}	Clinical effectiveness and safety of baricitinib for the treatment of juvenile idiopathic arthritis-associated uveitis or chronic anterior antinuclear antibody-positive uveitis: study protocol for an open-label, adalimumab active-controlled phase 3 clinical trial (JUVE-BRIGHT)
**Trial registration {2a and 2b}.**	EudraCT 2019-000119-10, January 4, 2019, https://www.clinicaltrialsregister.eu/ctr-search/search?query=2019-000119-10; NCT04088409, September 12, 2019, https://clinicaltrials.gov/ct2/show/NCT04088409.
**Protocol version**	Version 3 dated 31 May 2019
**Funding {4}**	This study was sponsored by Eli Lilly and Company, Indianapolis, IN, under license from Incyte Corporation.
**Author details {5a}**	^1^Athimalaipet V. Ramanan, ^2^Catherine M. Guly, ^3^Stuart Y. Keller, ^3^Douglas E. Schlichting, ^3^Stephanie de Bono, ^3^Ran Liao, ^4^Pierre Quartier ^1^Translational Health Sciences, University of Bristol, Bristol, UK; Department of Paediatric Rheumatology, University Hospitals Bristol NHS Foundation Trust, Bristol, UK; ^2^Bristol Eye Hospital, University Hospitals Bristol NHS Foundation Trust, Bristol, UK; ^3^Eli Lilly and Company, Indianapolis, IN, USA; ^4^Pediatric Immunology-Hematology and Rheumatology Unit, RAISE reference centre for rare diseases, Necker-Enfants Malades University Hospital, Assistance Publique-Hopitaux de Paris, IMAGINE Institute, Université de Paris, Paris, France.
**Name and contact information for the trial sponsor {5b}**	Eli Lilly and Company.
**Role of sponsor {5c}**	This study was designed jointly by consultant experts and representatives of the sponsor. Data will be collected by investigators and analyzed by the sponsor. Representatives of the sponsor were involved with drafting of the manuscript, including the decision to submit the manuscript for publication, and will be involved with collection, management, analysis, and interpretation of data.

## Introduction

### Background and rationale {6a}

Juvenile idiopathic arthritis (JIA) is defined as arthritis that has an onset in patients prior to 16 years of age, persisting for more than 6 weeks, and of unknown etiology. JIA belongs to a heterogeneous group of autoimmune diseases that represent the most common rheumatic condition of childhood and is estimated to affect 1 in 1000 children [1]. JIA accounts for approximately 55% of noninfectious pediatric uveitis and is the most common systemic disorder associated with uveitis in childhood [2]. Uveitis is more common in patients with JIA who are antinuclear antibody (ANA)-positive, have an early-onset disease, and have oligoarticular arthritis [3, 4]. Juvenile idiopathic arthritis-associated uveitis (JIA-uveitis) is typically anterior, chronic, bilateral, nongranulomatous, and asymptomatic [5]. Visual outcomes in JIA-uveitis have improved with current screening and treatment options; however, many patients fail to respond or do not achieve long-lasting remission with these treatments [6, 7]. Therefore, a critical need to develop more effective therapies for these patients remains.

Methotrexate (MTX) is well established as the immunosuppressive therapy of choice for patients with JIA-uveitis who have not responded adequately to topical corticosteroid therapy. Biologic disease-modifying antirheumatic drugs (bDMARDs) are initiated if there is intolerance of MTX or active uveitis despite an adequate trial of methotrexate therapy [8, 9]. Adalimumab, an anti-tumor necrosis factor alpha (TNFα) monoclonal antibody, is the only licensed biologic therapy for JIA-uveitis. Children with chronic ANA-positive uveitis without systemic features have a similar clinical course and treatment response to children with a diagnosis of JIA-uveitis. Therefore, the European Medicines Agency recommended that patients with chronic anterior ANA-positive uveitis without systemic symptoms be included in JIA-uveitis studies [10].

Biologic DMARDs, in particular the recently approved adalimumab, have led to significant improvements in JIA-uveitis treatment. The SYCAMORE study, a double-blind, randomized, placebo-controlled study that assessed the efficacy, safety, and cost-effectiveness of adalimumab plus MTX in JIA-uveitis, was completed in 2017. SYCAMORE was stopped early for efficacy after 90 of the planned 114 patients had been randomized [6]. Analysis of the primary endpoint (time to treatment failure) showed a positive treatment effect in favor of adalimumab (hazard ratio [HR] 0.25 [95% confidence interval [CI] 0.12–0.49]; *p* < 0.0001) [6]. The ADJUVITE study, a double-blind, randomized, placebo-controlled study, also supports the efficacy of adalimumab in patients with early-onset, chronic, JIA-associated or idiopathic anterior uveitis and an inadequate response to topical steroids and MTX, although the study was small [11]. The phase 2, single-arm (adaptive-trial), open-label APTITUDE trial evaluated the efficacy and safety of the fully humanized anti-interleukin (IL)-6R antibody, tocilizumab, with MTX in anti-TNF refractory JIA-uveitis. While the trial did not pass the prespecified criterion based on the adaptive design, data showed that of 21 patients, 33% had a 2-step improvement in the level of inflammation (anterior chamber cells) at week 12 and a further 14% had a 1-step improvement at week 24 [12]. Despite the increasing usage of biologics in JIA-uveitis, many patients fail to respond to or do not achieve long-lasting remission with these medications. For example, treatment failure occurred in 27% of patients on adalimumab and MTX in the SYCAMORE trial [6]. Furthermore, during a 2-year treatment period, 40% of JIA-uveitis patients receiving adalimumab and 80% receiving the anti-TNFα monoclonal antibody, infliximab, did not achieve clinical remission [7].

Baricitinib is an oral selective Janus kinase (JAK) 1 and JAK2 inhibitor with less activity against the JAK family members, tyrosine kinase 2 and JAK3 [13]. Baricitinib modulates cytokine signaling pathways implicated in disease pathogenesis by partially inhibiting JAK1 and JAK2 enzymatic activity, resulting in reduced phosphorylation and activation of signal transducers and activators of transcription (STATs) and reduced inflammation, cellular activation, and proliferation of key immune cells [14]. Baricitinib demonstrated acceptable clinical safety and efficacy in adult patients with moderately to severely active rheumatoid arthritis (RA) in 5 completed phase 3 studies [15–19]. Baricitinib is approved for the treatment of moderately to severely active RA in adults in over 65 countries including countries of the European Union, Japan, and the USA. Studies in patients with JIA [20, 21] or systemic JIA [22] are ongoing.

The etiology and pathogenesis of JIA, JIA-uveitis, and ANA-positive uveitis are still poorly understood, but the diseases share several immunological abnormalities identified in RA [1]. The inflammatory synovitis in JIA is similar to that observed in RA. In addition, some studies have shown that levels of inflammatory cytokines elevated in adults with RA, such as IL-6 and TNFα, are also elevated in the synovial fluid and serum of patients with JIA, as well as the aqueous humor of patients with JIA-uveitis [23]. Inflammatory cytokines, such as IL-6, which transduces cell signaling through the JAK/STAT pathway [24], and TNF, whose expression is reduced by inhibition of JAK1 and JAK2, are considered to be associated with the pathology of JIA-uveitis and ANA-positive uveitis [23, 25]. In addition to promising results observed with baricitinib treatment in patients with RA, baricitinib may inhibit multiple JAK/STAT-dependent cytokine pathways associated with JIA-uveitis and ANA-positive uveitis pathogenesis and provide a novel therapeutic approach to disease management.

### Objectives {7}

The aim of the ongoing multicenter, open-label, active-controlled study, JUVE-BRIGHT, is to evaluate the efficacy and safety of oral baricitinib administered once daily (QD) to pediatric patients with JIA-uveitis or chronic ANA-positive uveitis without systemic features who had an inadequate response to topical steroids, and MTX or bDMARDs [26].

### Trial design {8}

As results of the SYCAMORE study now make a placebo-controlled study ethically and logistically challenging, this uveitis study has been planned with an alternative design to study baricitinib as a treatment for JIA-uveitis and chronic ANA-positive uveitis. An open-label Bayesian design has been used for this study to maximize the benefit/risk balance for trial participants and overcome the statistical inference challenge for small sample size studies [27]. Two interim futility analyses have been embedded in the study design to ensure there is potential for efficacy with this investigational product. A Bayesian analysis will be conducted at interim analyses and the end of the study. The futility criteria and study success criteria were prespecified, and the Bayesian posterior probability will be calculated and used for decision-making.

This study includes adalimumab in the form of an active-control reference arm. The data from patients treated with adalimumab will be used for assay sensitivity and assay validation.

## Methods: participants, interventions, and outcomes

### Study setting

This study will be conducted at centers in France, Germany, Italy, Spain, and the UK [26] offering combined pediatric rheumatology/ophthalmology services.

### Eligibility criteria

#### Inclusion criteria

Patients are eligible to enroll in the study if they meet all of the following criteria at screening and at baseline:

1. Are at least 2 years and <18 years of age
2. Have a diagnosis of JIA-uveitis or chronic ANA-positive uveitis without systemic features
3. Have active anterior uveitis, defined as cellular infiltrate in the anterior chamber of SUN criteria grade ≥1+ at visit 1 (screening) and visit 2 (potential randomization), despite prior treatment with topical steroid therapy and MTX
4. Have an inadequate response or intolerance to MTX (minimum dose of 10 mg/m^2^/week, with a maximum dose of 25 mg/m^2^/week). Patients considered to have inadequate response must have received MTX for at least 12 weeks before an inadequate response may be determined and must have been on a stable dose for at least 4 weeks prior to screening if continuing MTX therapy during the study
5. Are receiving topical corticosteroid eye drops at a stable dose for at least 2 weeks prior to screening (maximum of 4 drops/day per eye at screening)
6. Parent or legal guardian and the patient (as appropriate) must sign their consent and assent, respectively
7. Male or nonpregnant, nonbreastfeeding female patients
  - Patients of child-bearing potential must agree to remain abstinent or use effective contraception practices throughout trial participation

#### Key exclusion criteria

Patients will be excluded from study enrollment if they meet any of the following criteria:

1. Have uveitis without a diagnosis of JIA or chronic anterior uveitis without positive ANA
2. Have a history or presence of any autoimmune inflammatory condition other than JIA, such as Crohn’s disease or ulcerative colitis
3. Have any contraindications to adalimumab as addressed in local product labeling or local clinical practice that would preclude the patient from participating in this study
  - Exception: Patients who have had an inadequate response or intolerance to bDMARDs (bDMARD-IR) with a contraindication to adalimumab may be enrolled, as they will be assigned to baricitinib

4. Have increased intraocular pressure ≥25 mm Hg or that required treatment, including increases in medications, surgery, or hospitalization, within 4 weeks prior to baseline that, in the opinion of the investigator, would pose an unacceptable risk to the patient if participating in the study
5. Have had intraocular surgery within the 3 months prior to screening (such as for cataract[s], glaucoma, or vitrectomy)
6. Have had symptomatic herpes zoster infection within 12 weeks prior to baseline
7. Have a history of multidermatomal herpes zoster, or complicated herpes zoster (e.g., ocular or motor nerve involvement or disseminated herpes zoster such as systemic infection)
8. Have a history of a venous thromboembolic event (VTE) or are considered at high risk of VTE as deemed by the investigator
9. Have body temperature ≥38°C (100.5°F) at baseline (visit 2)
10. Have initiated or changed dosage of concomitant mycophenolate mofetil or csDMARDs (other than MTX) within 4 weeks prior to screening (such as, but not limited to, hydroxychloroquine, sulfasalazine, gold salts, cyclosporine, or azathioprine)
11. Are currently receiving concomitant treatment with combination of >2 csDMARDs (including MTX)
12. Have received prior bDMARDs for any indication <1 week prior to screening for anakinra, <4 weeks prior to screening for TNF inhibitors (e.g., etanercept, infliximab, certolizumab, adalimumab, golimumab), other IL-1 inhibitors, IL-6 inhibitors (e.g., tocilizumab), or abatacept, and <6 months prior to screening for rituximab
13. Have received an unstable dose of analgesics, including NSAIDs, within 1 week prior to visit 2
14. Have received treatment with any parenteral corticosteroid administered by intraarticular, intramuscular, or intravenous injection within 4 weeks prior to visit 2
15. Are using oral corticosteroids at average daily doses >10 mg/day or 0.2 mg/kg/day prednisone equivalent, whichever is less, or have done so within 2 weeks prior to screening. If continuing oral corticosteroids, must be on a stable dose for 4 weeks prior to baseline
16. Have received a depot periocular, periocular, or intraocular corticosteroid injection within 30 days prior to visit 2
17. Have received an intraocular steroid implant within 6 months (e.g., dexamethasone intravitreal implant) or 18 months (e.g., fluocinolone acetonide intravitreal implant) prior to visit 2
18. Have received intraocular disease-modifying agents, including anti-vascular endothelial growth factor injections, for 30 days prior to visit 2
19. Are being treated with a strong organic anion transporter 3 inhibitor, such as probenecid, that cannot be discontinued for the duration of the study
20. Have been treated with a JAK inhibitor
21. Have commenced thyroxine therapy or changed dosage within 12 weeks prior to baseline or have thyroid-stimulating hormone levels outside the laboratory’s reference range
22. Discontinued within 30 days of study entry from any other clinical study involving an investigational product or any other type of medical research judged not to be scientifically or medically compatible with this study
  - If the previous investigational product has a long half-life, 5 half-lives or 30 days (whichever is longer) should have passed

### Who will take informed consent? {26a}

At screening, the investigator is responsible for ensuring that all parents or legal guardians and patients (as appropriate) provide written informed consent and assent, respectively.

### Additional consent provisions for collection and use of participant data and biological specimens {26b}

Consent includes the permission for collection and use of data and biological specimens.

### Interventions

#### Explanation for the choice of comparators {6b}

The JIA-uveitis patient population is not large (an estimated 1 in 10,000 children are affected) [9]. Most patients are first treated with MTX, and the biologic therapy adalimumab is approved for the treatment of this serious condition. Given the low incidence and prevalence of patients with JIA-uveitis who have tried and failed MTX, and the availability of an approved therapy for this condition, a traditional study with placebo control and frequentist design was not considered appropriate. The intent of this design is to minimize patient exposure to ineffective interventions (therefore, no placebo group was included, and interim efficacy analyses for the experimental therapy, baricitinib, were prespecified). The design allows continued treatment for patients if baricitinib shows potential efficacy and protects patients from unnecessary exposure to baricitinib if there is little potential for efficacy. Bayesian analyses will be conducted at interim time points and the end of the study (if the study is not stopped earlier).

#### Intervention description {11a}

This is a multicenter, open-label, active-controlled phase 3 study in patients with active JIA-uveitis or chronic ANA-positive uveitis without systemic features, despite prior treatment with topical steroid therapy, and MTX or bDMARDs. Patients aged 2 to <18 years will be treated with oral baricitinib QD (administered as tablets or oral suspension based on the age of the patient) at a fixed dose by age group (4 mg for patients ≥6 to <18 years of age and 2 mg for patients <6 years of age) or adalimumab (20 mg for patients <30 kg or 40 mg for patients weighing ≥30 kg) as a subcutaneous injection every 2 weeks.

#### Criteria for discontinuing or modifying allocated interventions {11b}

The baricitinib dose and formulation for an individual patient will not change during part A of this study. During part B (the open-label extension [OLE]), as patients age over 9 years old, they will receive baricitinib 4 mg at their next scheduled visit, and as patients age over 6 years old, they will have the option of receiving tablets instead of suspension. For patients who progress to the ≥18 years age group, their current dose will be maintained.

Adalimumab dose modifications are not permitted in patients whose body weight changes from <30 to ≥30 kg or from ≥30 to <30 kg during part A. The dose of adalimumab can be increased during part B if the patient has a sustained (2 or more visits) weight increase from <30 to ≥30 kg.

Patients receiving adalimumab who experience a treatment failure after at least 24 weeks of treatment may receive rescue therapy with baricitinib, which they will receive for the remainder of the study.

Patients who do not continue in the OLE will have a follow-up visit approximately 28 days after the last dose of the investigational product.

#### Strategies to improve adherence to interventions {11c}

The investigator is responsible for investigational product accountability, reconciliation, and record maintenance. Patient compliance with study medication will be assessed during the treatment period of part A and part B per the schedule of activities. Patients will be counseled by staff on the importance of taking the investigational product as prescribed, as appropriate.

#### Relevant concomitant care permitted or prohibited during the trial {11d}

The study allows for treatment with background conventional synthetic DMARDs (csDMARDs), corticosteroids, and/or nonsteroidal anti-inflammatory drugs (NSAIDs) at a stable dose, which must be recorded on the concomitant medication electronic case report form (eCRF).

#### Provisions for post-trial care {30}

Not applicable.

### Outcomes {12}

#### Primary endpoint

The choice of primary and secondary endpoints arose from work started in 2005, when the Standardization of Uveitis Nomenclature (SUN) Working Group provided a standardized nomenclature for uveitis, inflammation grading, and outcome measures [28]. This nomenclature has been reviewed by a working group of ophthalmologists and pediatric rheumatologists to create recommendations for reporting clinical outcomes in JIA-uveitis clinical studies [8].

The primary endpoint of the study is the proportion of patients with response at week 24. Response is defined according to the SUN criteria as a 2-step decrease in the level of inflammation (anterior chamber cells) or decrease to zero through week 24 in the eye most severely affected at baseline.

#### Secondary endpoints

The primary endpoint will be supported by key secondary outcomes to assess change in grade of cells in the anterior chamber from baseline, age-appropriate visual acuity, change in vitreous haze, grade of flare in the anterior chamber, length of time to inactive disease (as defined by SUN criteria), tapering of concomitant steroids, pediatric clinical outcomes assessments and patient-reported outcomes, and overall uveitis-related disability.

The following secondary endpoints will be assessed at each visit:

1. Change in SUN grade of cells in the anterior chamber through week 24 (part A) and through week 284 (part B) in the most severely affected eye
2. Change in SUN grade of cells in the anterior chamber through week 24 and week 284 in the less severely affected eye (if applicable)
3. In patients with bilateral uveitis disease at baseline: proportion of responders at weeks 24 and 284, defined according to the SUN criteria as a 2-step decrease in the level of anterior chamber cells in the most severely affected eye at baseline (or both eyes if the inflammation grade is the same in both eyes) and a 1-step decrease in the level of anterior chamber cells in the less severely affected eye at baseline
4. Change in visual acuity measured by age-appropriate logarithm of the minimum angle of resolution test through week 24
5. Change in vitreous haze through week 24 in each affected eye
6. Change in grade of flare in the anterior chamber through week 24 and week 284 in each affected eye
7. Change in overall uveitis-related disability:
  - Change in Patient Uveitis-related Disease Activity through week 24
  - Change in Patient Uveitis-related Improvement at week 12 and week 24
  - Change in Patient Arthritis Disease Activity through week 24
  - Change in Patient Arthritis Improvement at week 12 and week 24
  - Change in Ophthalmologist Uveitis-related Disease Activity through week 24
  - Change in Ophthalmologist Uveitis-related Improvement at week 12 and week 24
8. Proportion of patients with inactive anterior uveitis (using SUN definition) in each affected eye through week 24 and week 284
9. Time to inactive anterior uveitis disease (using SUN definition) in each affected eye
10. Proportion of patients who are able to taper concomitant corticosteroids
11. Time to treatment response: response is defined by a 2-step decrease in the level of inflammation (anterior chamber cells) or decrease to zero in the eye most severely affected at baseline
12. Proportion of responders at week 284
13. Pediatric American College of Rheumatology (PediACR) 30/50/70/90/100 response rates (for patients with JIA-uveitis)
14. Safety variables:
  - Adverse events (AEs), including serious adverse events
  - Permanent discontinuation of investigational product due to AEs
  - Temporary interruption of investigational product

### Participant timeline {13}

**Fig. 1:**
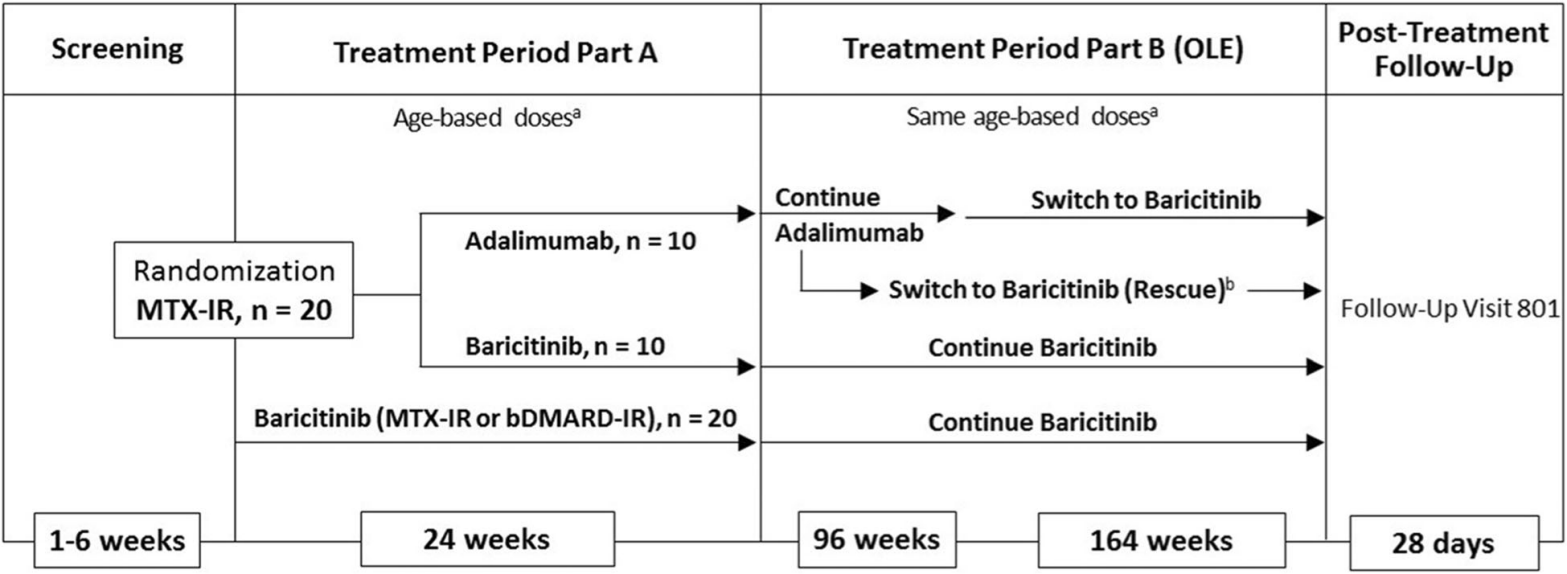
Schematic of trial design. ^a^Patients ≥6 to <12 years old assigned to baricitinib have the option of receiving the oral suspension or tablets. Patients >12 years old assigned to baricitinib will receive tablets. Patients assigned to adalimumab weighing <30 kg will receive 20 mg, and those ≥ 30 kg will receive 40 mg. ^b^Patients receiving adalimumab who experience a treatment failure after at least 24 weeks of treatment may receive rescue therapy with baricitinib. Rescued patients will receive baricitinib for the remainder of the study. bDMARD, biologic disease-modifying antirheumatic drug; MTX-IR, inadequate response or intolerance to methotrexate; OLE, open-label extension

In accordance with the SPIRIT 2013 checklist, Fig. 1 illustrates a schematic of the trial design. The study will be conducted in 2 parts. Part A will include the screening period and treatment period that includes open-label treatment with baricitinib or adalimumab up to week 24. Part B (the OLE) will provide treatment for up to 5 years for patients who complete part A. Patients receiving baricitinib during part A will continue to receive baricitinib during part B. Patients receiving adalimumab during part A will continue to receive adalimumab for an additional 96 weeks and will then switch to baricitinib for the remaining 164 weeks of part B. The average duration for treatment of JIA-uveitis with adalimumab in current clinical practice is 2 years [29]. Switching from adalimumab to baricitinib after 96 weeks will allow the effect of this switch to be examined in the context of a clinical trial, at a point where the positive benefit/risk of adalimumab in JIA-uveitis has been established.

### Sample size {14}

The study will enroll at least 20 and up to 40 patients who have had an inadequate response or intolerance to MTX (MTX-IR) and/or bDMARDs (bDMARD-IR) as follows: approximately 30 patients in total will be treated with baricitinib, and at least 10 patients will be treated with adalimumab. At least 20 patients who are MTX-IR (but not bDMARD-IR) will be randomized in a 1:1 ratio to either open-label baricitinib or adalimumab; at least 10 patients will be randomized to baricitinib, and at least 10 patients will be randomized to adalimumab (Fig. 1). Approximately 20 additional patients who are MTX-IR or bDMARD-IR will be treated with baricitinib. Patients who are bDMARD-IR will only receive open-label baricitinib to avoid retreatment with a bDMARD. With a total sample size of 30 in the baricitinib arm, and assuming an observed response rate of 66.7%, the study will be able to detect a true baricitinib treatment response rate of >57% with >80% probability. The observed response rate of 66.7% is based on the assumption that 20 out of 30 patients in the baricitinib arm will achieve the primary endpoint. The response rate threshold of 57% was chosen to match the response rate observed for patients with JIA-uveitis treated with adalimumab in the SYCAMORE study [6].

### Recruitment {15}

The sponsor or its designee is responsible for the central recruitment strategy for patients. Individual investigators may have additional local requirement and processes.

### Assignment of interventions: allocation

#### Sequence generation {16a}

Assignment to treatment groups will be determined by a computer-generated random sequence using an interactive web-response system (IWRS).

#### Concealment mechanism {16b}

The IWRS will be used to assign the investigational product to each patient.

#### Implementation {16c}

Site personnel will confirm that they have located the correct packages by entering a confirmation number found on the packages into the IWRS before dispensing to the patient.

### Assignment of interventions: blinding

#### Who will be blinded {17a}

Not applicable; this is an open-label study.

#### Procedure for unblinding if needed {17b}

Not applicable; no blinding occurs as part of this open-label study.

### Data collection and management

#### Plans for assessment and collection of outcomes {18a}

Table 1 lists key study assessments and procedures with their timing. At screening, the parent or legal guardian will sign the informed consent form and the patient will sign the assent form (as appropriate) per local requirements prior to any study assessments, examinations, or procedures being performed. All screening procedures will be performed as outlined in Table 1, and patients who meet all of the inclusion and none of the exclusion criteria will continue to baseline. At baseline, study eligibility for each patient will be reviewed, based on all inclusion and exclusion criteria and laboratory test results. Patients who meet all criteria will proceed to the subsequent period. Laboratory samples will be collected at baseline and all assessments should be completed before the patient takes the first dose of investigational product. Procedures will be performed during treatment period part A as outlined in Table 1. A similar set of procedures will be carried out during treatment period part B, the long-term OLE. All assessments utilized in this study are standard, widely used, and generally recognized as reliable, accurate, and relevant, except for the Uveitis-related Disease Activity and Improvement and Arthritis Disease Activity and Improvement scales, which are novel instruments developed by the sponsor.

#### Plans to promote participant retention and complete follow-up {18b}

Patients who complete the study or discontinue early from the study will have a posttreatment safety follow-up visit (visit 801) approximately 28 days after the last dose of the investigational product. Patients who have received at least 1 dose of the investigational product and terminate participation early must have an early termination visit (ETV). If the ETV occurs on the same day as the scheduled visit, any assessments/procedures conducted during the scheduled visit should not be repeated for a separate ETV.

#### Data management {19}

An electronic data capture (EDC) system will be used in this study for the collection of CRF data. Clinical outcome assessment (COA) data will be collected by the patient/caregiver/investigator site personnel via a paper source document and will be transcribed by the investigator personnel in the EDC system. Electronic COA (eCOA) data will be directly recorded by the patient/caregiver/investigator site personnel into an instrument. To ensure accurate, complete, and reliable data, the sponsor or its representative will provide instructional material to the study sites, as appropriate, provide sponsor start-up training, make periodic visits to study sites, be available for consultation and remain in contact with the study site personnel, and review and verify data reported to detect potential errors. The sponsor or its representatives will periodically check a sample of patient data recorded against source documents at the study site.

**Table 1:** Study visits and selected assessments

	Screening		Treatment period part A							Early termination	Posttreatment follow-up
Visit #	V1	V1^**a**^	V2^**a**^ Baseline	V3	V4	V5	V6	V7	V8	ETV^**b**^	V801^**c**^
**Study week**			**W0**	**W4**	**W8**	**W12**	**W16**	**W20**	**W24**	**Any week**	
**Study day (approximately)**	**−42 to −1**		**0**	**28**	**56**	**84**	**112**	**140**	**168**	**Any day**	**28 ± 5 days after last dose**
**Visit window (days)**				**±4**							
Informed consent and assent	X										
Demographics	X										
Physical examination^d^	X										
Symptom-directed physical examination^d^			X	X	X	X	X	X	X	X	X
Height			X			X			X	X	
Weight			X	X	X	X	X	X	X	X	X
Occipital frontal circumference measurement in children up to 3 years of age			X	X^e^						X	X
Vital signs (blood pressure, pulse, temperature)			X	X	X	X	X	X	X	X	X
JIA diagnosis (ILAR criteria)	X										
Previous JIA and uveitis therapy	X										
Visual acuity (LogMAR)			X	X	X	X	X	X	X	X	X
Slit-lamp examination of the retina and optic disc			X	X	X	X	X	X	X	X	X
Optical coherence tomography			X	X	X	X	X	X	X	X	X
Slit-lamp examination for anterior chamber cells and flare assessment	X		X	X	X	X	X	X	X	X	X
Slit-lamp examination for assessment of vitritis and vitreous haze	X		X	X	X	X	X	X	X	X	X
Cataract scoring^f^			X	X	X	X	X	X	X	X	X
IOP using I-Care tonometry, Goldmann tonometry, or Tono-Pen			X	X	X	X	X	X	X	X	X
Concomitant medications	X		X	X	X	X	X	X	X	X	X
Joint assessment	X		X	X	X	X	X	X	X	X	X
Physician’s Global Assessment of Disease Activity	X		X	X	X	X	X	X	X	X	X
Patient Uveitis-related Disease Activity^g^	X		X	X	X	X	X	X	X	X	X
Patient Uveitis-related Improvement^g^						X			X	X	X
Patient Arthritis Disease Activity^g^	X		X	X	X	X	X	X	X	X	X
Patient Arthritis Improvement^g^						X			X	X	X
Ophthalmologist Uveitis-related Disease Activity	X		X	X	X	X	X	X	X	X	X
Ophthalmologist Uveitis-related Improvement						X			X	X	X
CHAQ^h^			X	X	X	X	X	X	X	X	X
CHQ-PF50^h^			X			X			X	X	X
Morning stiffness duration^g^			X	X	X	X	X	X	X	X	X
SPARCC Enthesitis Index^i^			X	X	X	X	X	X	X	X	X
Clinical sacroiliitis^i^			X	X	X	X	X	X	X	X	X
Back mobility (Schober’s test)^i^			X	X	X	X	X	X	X	X	X
PASI^j^			X	X	X	X	X	X	X	X	X
hsCRP	X		X	X	X	X	X	X	X	X	X
ESR^k^			X	X	X	X	X	X	X	X	X
HLA-B27					X						
RF and ACPA	X										
Antinuclear antibodies			X						X	X	
Clinical chemistry^l^	X		X	X	X	X	X	X	X	X	X
Hematology	X		X	X	X	X	X	X	X	X	X
Urinalysis	X		X			X			X	X	X
Iron studies (iron, TIBC, and ferritin)			X			X			X	X	X
Fasting lipid panel			X			X			X	X	X
IgA, IgG, IgM			X			X			X	X	X

^a^Baseline laboratory samples should be taken before administration of the investigational product

^b^ETV occurs if the patient terminates participation early. If the ETV occurs on the same day as the scheduled visit, any assessments/procedures conducted during the scheduled visit should not be repeated for a separate ETV

^c^Patients who complete the study or discontinue early from the study will have a posttreatment safety follow-up visit (V801) approximately 28 days after the last dose of investigational product

^d^One physical examination will be performed at visit 1. All subsequent physical examinations may be symptom-directed

^e^Occipital frontal circumference measurement is required every 3 months for patients under 3 years of age, but is no longer required once the patient reaches 3 years of age

^f^For patients with cataracts at baseline, or who develop cataracts during the study

^g^Patient-reported and caregiver-reported questionnaires will be administered on paper at the site and are recommended to be completed prior to any clinical examinations

^h^Caregiver-reported questionnaires will be administered via an on-site electronic Clinical Outcome Assessment device and are recommended to be completed prior to any clinical assessments

^i^For patients with enthesitis-related juvenile idiopathic arthritis or JPsA

^j^For patients with JPsA

^k^Performed locally. To be drawn prior to dosing early in the visit

^l^Clinical chemistry will include eGFR

*ACPA*, anti-citrullinated protein antibodies; *CHAQ*, Childhood Health Assessment Questionnaire; *CHQ-PF50*, Child Health Questionnaire-Parent Form 50; *eGFR*, estimated glomerular filtration rate; *ESR*, erythrocyte sedimentation rate; *ETV*, early termination visit; *HLA-B27*, human leukocyte antigen-B27; *hsCRP*, high-sensitivity C-reactive protein; *Ig*, immunoglobulin; *ILAR*, International League of Associations for Rheumatology; *IOP*, intraocular pressure; *JIA*, juvenile idiopathic arthritis; *JPsA*, juvenile psoriatic arthritis; *LogMAR*, logarithm of the minimum angle of resolution; *PASI*, Psoriasis Area and Severity Index; *RF*, rheumatoid factor; *SPARCC*, Spondyloarthritis Research Consortium of Canada; *TIBC*, total iron-binding capacity; *V*, visit; *W*, week

#### Confidentiality {27}

Patient confidentiality will be maintained in accordance with the Data Privacy Statement in the Informed Consent Form.

#### Plans for collection, laboratory evaluation, and storage of biological specimens for genetic or molecular analysis in this trial/future use {33}

A whole blood sample will be collected for pharmacogenetic analysis at week 8 where local regulations allow. Blood for nonpharmacogenetic biomarker research will be collected at weeks 0, 12, and 24 where local regulations allow.

Samples will be retained at a facility selected by the sponsor for a maximum of 15 years after the last patient visit for the study, or for a shorter period if local regulations require and ethical review boards impose shorter time limits. Any samples remaining after 15 years will be destroyed.

### Statistical methods

#### Statistical methods for primary and secondary outcomes {20a}

The analysis will be carried out according to the predefined statistical analysis plan. A Bayesian analysis will determine if the study should be considered a positive study or a negative study. The study will be considered positive if the posterior probability is at least 80% that the baricitinib treatment response is >57%; otherwise, the study will be considered negative. Response will be evaluated after 30 patients complete 24 weeks of treatment. Descriptive response rates will be evaluated in adalimumab-treated patients. All safety data will be descriptively summarized using corresponding populations. Efficacy and health outcome endpoints will be summarized using descriptive statistics. Point estimates and 95% CIs will be computed.

#### Interim analyses {21b}

Two interim analyses will be performed to determine if the study should be stopped for futility when 10 and 20 baricitinib-treated patients have completed 24 weeks of treatment. At each interim analysis, the posterior probability of the treatment response rate being lower than 40% will be calculated, and the study will be stopped if this probability is >75%; otherwise, the study will continue. If the study is not stopped for futility, the enrollment will continue to 30 baricitinib-treated patients.

**Fig. 2:**
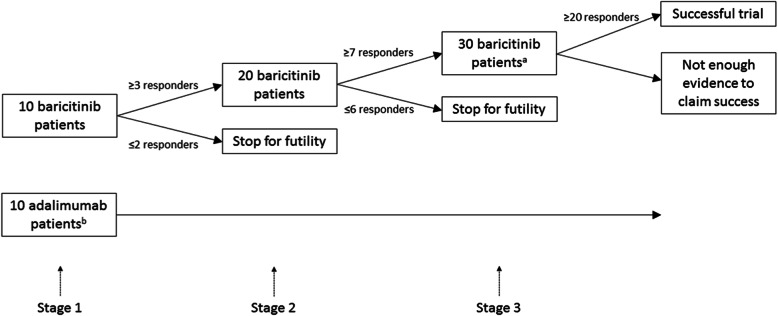
Adaptive design for the efficacy of baricitinib-treated patients. ^a^Includes a minimum of 10 MTX-refractory/intolerant patients. ^b^All MTX-refractory/intolerant patients, naïve to biologics. MTX, methotrexate

Given the proposed overall sample size and timing of interim analyses, the above Bayesian decision rules can be translated to correspond to the following as shown in Fig. 2:

- At the first interim analysis, the study will be stopped if 2 or fewer of the 10 patients are responders.
- If the study continues, at the second interim analysis, the study will be stopped if 6 or fewer of the 20 patients are responders.
- If the study continues, at the final analysis, the study objective will be successfully met (i.e., a positive study) if at least 20 of the 30 patients are responders.

A data monitoring committee (DMC) consisting of specialists external to the sponsor will oversee the conduct of the study following rules defined in the DMC charter. The DMC will review and evaluate planned interim analyses.

#### Methods for additional analyses (e.g., subgroup analyses) {20b}

An exploratory analysis will evaluate response rates in MTX-IR, adalimumab-treated patients and MTX-IR, baricitinib-treated patients.

#### Methods in analysis to handle protocol non-adherence and any statistical methods to handle missing data {20c}

The proportion of missing data will be presented, including details of the reason for missingness. For each patient who discontinues treatment before completing the 24-week assessment of the primary outcome, a clinical decision will be made by a clinical endpoint evaluation committee composed of study investigators as to whether the patient should be classified as a responder or nonresponder.

### Plans to give access to the full protocol, participant-level data, and statistical code {31c}

Lilly provides access to all individual participant data collected during the trial, after anonymization, with the exception of pharmacokinetic or genetic data. Data are available to request 6 months after the indication studied has been approved in the USA and EU and after primary publication acceptance, whichever is later. No expiration date of data requests is currently set once data are made available. Access is provided after a proposal has been approved by an independent review committee identified for this purpose and after receipt of a signed data sharing agreement. Data and documents, including the study protocol, statistical analysis plan, clinical study report, and blank or annotated case report forms, will be provided in a secure data sharing environment. For details on submitting a request, see the instructions provided at www.vivli.org**.**

### Oversight and monitoring

#### Composition of the data monitoring committee, its role, and reporting structure {21a}

A DMC consisting of specialists external to the sponsor will oversee the conduct of the study following rules defined in the DMC charter. The DMC will review and evaluate planned interim analyses.

#### Adverse event reporting and harms {22}

An independent, masked clinical event committee will adjudicate potential cardiovascular events, venous thrombotic events, and noncardiovascular deaths.

#### Frequency and plans for auditing trial conduct {23}

The study may be audited by Eli Lilly and Company or its representatives and/or regulatory agencies at any time with investigators being notified beforehand.

#### Plans for communicating important protocol amendments to relevant parties (e.g., trial participants, ethical committees) {25}

Investigators, ethical review boards, and regulators will be notified of any important modifications to the protocol.

#### Dissemination plans {31a}

The results of this study will be submitted to a peer-reviewed journal 18 months after the last patient visit.

## Discussion

The aim of JUVE-BRIGHT is to evaluate the efficacy and safety of baricitinib when administered QD to pediatric patients with JIA-uveitis or ANA-positive uveitis who have had an inadequate response to topical steroids, and MTX or bDMARDs. The ever increasing number of new therapies makes clinical trials in children with rheumatic diseases increasingly more difficult due to the smaller number of children available for such studies. Head-to-head or noninferiority studies are not possible in pediatric rheumatic diseases due to the rarity of diseases and the associated sample sizes that would be required. Historical comparisons with other placebo-controlled trials do not address the key issue that access to care and therapeutic paradigms change every couple of years. This trial is novel in its attempt to have a reference arm of a standard of care biological agent which helps address issues important to patients/caregivers and clinicians. Furthermore, it is important that phase 3 studies address the key clinical questions that patients/caregivers and clinicians face, including which therapy is the right one for them? The current study assesses patient-related outcomes and thus will provide useful data to help answer such clinical questions.

The inclusion of MTX-IR patients was driven by physician and patient preference for an alternative oral therapeutic option for pediatric patients with JIA-uveitis who are MTX-IR [30]. Adalimumab is the only approved therapy for JIA-uveitis and is included in this study to provide an active comparator arm. In adult RA, baricitinib provides superior efficacy compared to adalimumab in patients who have an inadequate response to MTX [18]. Gene expression profiling showed significant differences between baricitinib and adalimumab treatments in adult RA. Baricitinib and adalimumab modulated JAK/STAT or complement pathways, respectively, and the drugs had opposite effects on interferons, indicating different and possibly complementary mechanisms of action of each targeted therapy [31]. Identification of JIA-uveitis treatments with differing mechanisms of action may continue to reduce the remaining unmet need. Furthermore, an oral treatment option such as baricitinib may be preferable to injectable bDMARDs for both patients and caregivers.

JIA-uveitis is a potentially sight-threatening condition and thus carries a considerable risk of morbidity, so new and alternative treatment options are needed [32]. However, novel trial designs are also needed to assess the effect of potential therapies. A conventional frequentist power-driven, double-blind, placebo-controlled study such as the SYCAMORE study, with its associated sample size of 90 [6], is ethically and logistically challenging considering the low incidence of JIA-uveitis. Minimization or elimination of exposure to placebo, maximizing statistical power, and generation of meaningful data to patients and prescribers in a relatively small patient population drove the consideration of novel designs beyond traditional placebo-controlled, frequentist methodology. A controlled, open-label, Bayesian design with interim assessments for futility has been implemented for the assessment of baricitinib in the treatment of JIA-uveitis. As this study formed part of a pediatric investigational plan in Europe, the study design and analysis plan needed to be approved by the PDCO as well as national regulatory agencies.

The Bayesian design is based on a strong foundation in statistical methodology and is considered a valid approach endorsed by regulatory agencies [33, 34]. For baricitinib-treated JIA-uveitis, the intent of this design is to continue treatment for patients if baricitinib shows potential efficacy and to prevent unnecessary exposure to baricitinib if there is little chance of demonstrating clinically meaningful efficacy. Bayesian analyses will be conducted at interim time points and the end of the study (if the study is not stopped earlier). In the SYCAMORE study, significantly more patients treated with adalimumab showed a response at 3 months compared with patients treated with placebo [6]. The time to treatment response during the SYCAMORE study informed the selection of 24 weeks as the time point for the primary endpoint in JUVE-BRIGHT.

This study will also be important to describe the safety profile of baricitinib in children with JIA-uveitis. The study will carefully monitor adverse events observed with baricitinib treatment in adults with RA, such as herpes zoster and thrombosis [35], in the pediatric JIA-uveitis and ANA-positive uveitis patient population included in this trial.

The efficacy, safety, and tolerability data from this novel study will establish an understanding of the benefit/risk relationship for baricitinib in pediatric patients with JIA-uveitis and ANA-positive uveitis with a high unmet need.

## Trial status

At the time of manuscript submission, this trial was open at 15 hospital sites and had recruited 6 patients. Protocol version number and date: version 3 dated 31 May 2019. This trial began recruitment on 16 October 2019 with recruitment expected to end near 28 January 2022.

## Data Availability

Lilly provides access to all individual participant data collected during the trial, after anonymization, with the exception of pharmacokinetic or genetic data. Data are available to request 6 months after the indication studied has been approved in the USA and EU and after primary publication acceptance, whichever is later. No expiration date of data requests is currently set once data are made available. Access is provided after a proposal has been approved by an independent review committee identified for this purpose and after receipt of a signed data sharing agreement. Data and documents, including the study protocol, statistical analysis plan, clinical study report, and blank or annotated case report forms, will be provided in a secure data sharing environment. For details on submitting a request, see the instructions provided at www.vivli.org**.**

## Abbreviations

AE: Adverse event
ANA: Antinuclear antibody
bDMARD-IR: Inadequate response or intolerance to bDMARDs
bDMARD: Biologic disease-modifying antirheumatic drug
CI: Confidence interval
COA: Clinical outcome assessment
csDMARD: Conventional synthetic DMARD
DMC: Data monitoring committee
eCOA: Electronic clinical outcome assessment
eCRF: Electronic case report form
EDC: Electronic data capture
EMA: European Medicines Agency
ETV: Early termination visit
HR: Hazard ratio
IL: Interleukin
IWRS: Interactive web-response system
JAK: Janus kinase
JIA: Juvenile idiopathic arthritis
JIA-uveitis: JIA-associated uveitis
MTX: Methotrexate
MTX-IR: Inadequate response or intolerance to methotrexate
NSAID: Nonsteroidal anti-inflammatory drug
OLE: Open-label extension
PediACR: Pediatric American College of Rheumatology
PDCO: The EMA Paediatric Committee
QD: Once daily
RA: Rheumatoid arthritis
STAT: Signal transducer and activator of transcription
SUN: Standardization of Uveitis Nomenclature
TNFα: Tumor necrosis factor alpha
VTE: Venous thromboembolic event
